# Urine cell cycle arrest biomarkers distinguish poorly between transient and persistent AKI in early septic shock: a prospective, multicenter study

**DOI:** 10.1186/s13054-020-02984-6

**Published:** 2020-06-01

**Authors:** Dimitri Titeca-Beauport, Delphine Daubin, Ly Van Vong, Guillaume Belliard, Cédric Bruel, Sami Alaya, Karim Chaoui, Maud Andrieu, Isabelle Rouquette-Vincenti, Frederic Godde, Michel Pascal, Momar Diouf, Christophe Vinsonneau, Kada Klouche, Julien Maizel

**Affiliations:** 1 BoReal Study Group, Medical Intensive Care Unit and EA7517, Amiens University Hospital, F-80054 Amiens, France; 2 Department of Intensive Care Medicine, Lapeyronie University Hospital, Montpellier, France; 3 Intensive Care Unit, Groupe Hospitalier Sud Ile de France, 270 avenue Marc Jacquet, 77000 Melun, France; 4 Medical-Surgical Intensive Care Unit, Centre Hospitalier de Bretagne Sud, Lorient, France; 5 Medical and Surgical Intensive Care Unit, Groupe Hospitalier Paris Saint Joseph, Paris, France; 6 Intensive Care Unit, Centre Hospitalier Général, 13300 Salon-de-Provence, France; 7 Intensive Care Unit, Jean Rougier Hospital, 335, rue du Président Wilson, 46000 Cahors, France; 8 Medical and Surgical Intensive Care Unit, Centre Hospitalier de Dax-Côte d’Argent, Dax, France; 9 Department of Anesthesia and Intensive Care, Princess Grace Hospital, Avenue Pasteur, Monaco (Principality), Monaco; 10 Département de Réanimation Polyvalente, Centre Hospitalier Avranches-Granville, Granville, France; 11 Intensive Care Unit, Centre Hospitalier de Mont De Marsan, 40000 Mont-de-Marsan, France; 12 Clinical Research and Innovation Directorate, Amiens University Hospital, Amiens, France; 13 BoReal Study Group, Intensive Care Unit, Hôpital de Bethune, 62408 Bethune, France

**Keywords:** Acute kidney injury, Septic shock, Biomarkers, Recovery

## Abstract

**Background:**

The urine biomarkers tissue inhibitor of metalloproteinases-2 (TIMP-2) and insulin-like growth factor-binding protein 7 (IGFBP7) have been validated for predicting and stratifying AKI. In this study, we analyzed the utility of these biomarkers for distinguishing between transient and persistent AKI in the early phase of septic shock.

**Methods:**

We performed a prospective, multicenter study in 11 French ICUs. Patients presenting septic shock, with the development of AKI within the first 6 h, were included. Urine [TIMP-2]*[IGFBP7] was determined at inclusion (0 h), 6 h, 12 h, and 24 h. AKI was considered transient if it resolved within 3 days. Discriminative power was evaluated by receiver operating characteristic (ROC) curve analysis.

**Results:**

We included 184 patients, within a median [IQR] time of 1.0 [0.0–3.0] h after norepinephrine (NE) initiation; 100 (54%) patients presented transient and 84 (46%) presented persistent AKI. Median [IQR] baseline urine [TIMP-2]*[IGFBP7] was higher in the persistent AKI group (2.21 [0.81–4.90] (ng/ml)^2^/1000) than in the transient AKI group (0.75 [0.20–2.12] (ng/ml)^2^/1000; *p* < 0.001). Baseline urine [TIMP-2]*[IGFBP7] was poorly discriminant, with an AUROC [95% CI] of 0.67 [0.59–0.73]. The clinical prediction model combining baseline serum creatinine concentration, baseline urine output, baseline NE dose, and baseline extrarenal SOFA performed well for the prediction of persistent AKI, with an AUROC [95% CI] of 0.81 [0.74–0.86]. The addition of urine [TIMP-2]*[IGFBP7] to this model did not improve the predictive performance.

**Conclusions:**

Urine [TIMP-2]*[IGFBP7] measurements in the early phase of septic shock discriminate poorly between transient and persistent AKI and do not improve clinical prediction over that achieved with the usual variables.

**Trial registration:**

NCT02812784

## Background

Sepsis is the most common factor contributing to acute kidney injury (AKI) in critically ill patients, accounting for up to 40% of cases [1, 2]. A recent study estimated that 68% of patients with sepsis had AKI at admission, with 40% presenting severe AKI and 27% subsequently undergoing renal replacement therapy (RRT) during intensive care unit (ICU) stay [3]. The development of AKI is associated with higher mortality and longer hospital stays.

Distinguishing between transient and persistent AKI is a key goal in clinical practice, as the early recognition of transient AKI allows a conservative strategy with respect to RRT initiation, whereas the early recognition of persistent AKI can help to guide fluid management, preventing deleterious fluid overload [4, 5]. The classical distinction between prerenal (functional) and structural AKI is of little relevance in critically ill patients [6], particularly in those with sepsis-associated AKI, which is more closely related to renal microcirculatory dysfunction than to a decrease in global renal blood flow [7, 8]. Most recent studies assessing the ability of urine biochemistry and derived indices to discriminate between transient and persistent AKI in critically ill patients have reported conflicting findings and relatively limited performances incompatible with use in clinical practice [9–15].

The product of the urinary concentrations of tissue inhibitor of metalloproteinases-2 (TIMP-2) and insulin-like growth factor-binding protein 7 (IGFBP7) has been validated for use in the early identification of sepsis-associated AKI and for risk stratification in this context [16–18]. However, only two small studies have assessed the ability of [TIMP-2]*[IGFBP7] to predict renal recovery in critically ill patients [19, 20]. It remains to be determined whether these biomarkers can discriminate effectively between transient and persistent sepsis-associated AKI.

The objective of this study was to determine whether [TIMP-2]*[IGFBP7] could distinguish between transient and persistent AKI in the early phase of septic shock. The secondary objective was to evaluate the potential usefulness of these biomarkers in combination with routinely available clinical data.

## Methods

### Patients and setting

This prospective study was conducted in 11 medical and multisystem ICUs between September 2015 and April 2017. The study protocol was approved by the North-West II Institutional Review Board (CPP Nord Ouest II, 2015-A01392-47). The next of kin of the patients were informed and gave consent for the patient’s participation in the study.

Adult patients admitted for septic shock according to the Third International Consensus Criteria [21] and presenting AKI within 6 h of the introduction of vasopressors and a bladder catheter were included in this study. The exclusion criteria were AKI requiring immediate RRT according to the attending physician, anuria, severe chronic kidney disease (CKD) (defined as a glomerular filtration rate < 30 ml/min per 1.73 m^2^), obstructive AKI, pregnancy, cardiocirculatory arrest, life expectancy < 48 h, Child C cirrhosis, prior AKI during the current hospital stay, kidney transplantation, and being a ward of court or under legal guardianship or having no social security cover.

### Study endpoint

The main objective of this study was to determine whether urine TIMP-2-IGFBP7 concentrations can be used to distinguish between transient and persistent AKI in the early phase of septic shock.

### Study procedure

All patients were treated according to the 2016 Surviving Sepsis campaign guidelines, including the following, within the first few hours: broad-spectrum antibiotics; infection source control, including surgical procedures if necessary; initial fluid resuscitation with 30 ml/kg crystalloids; and checking of fluid responsiveness over the next few days to prevent fluid overload.

H0 (baseline) was defined by the inclusion time and corresponded to the time at which the first urine sample was collected. Fresh urine samples were collected via a bladder catheter at inclusion (H0) and then 6 h, 12 h, and 24 h after inclusion. The urine was centrifuged, and the supernatant was frozen at − 80 °C until analysis. The urine samples were analyzed blindly to clinical or biological data. We calculated [TIMP-2]*[IGFBP7] with a 100-μl sample of thawed urine and the NephroCheck® Test (Astute Medical Inc., San Diego, CA, USA). The concentrations of the two biomarkers were multiplied, and the result was divided by 1000, to yield a risk value ranging from 0.02–135 (ng/ml)^2^/1000. The inter-assay coefficients of variation (CVs) provided by the manufacturer were between 8.1 and 11.4% for TIMP-2 and between 6.6 and 7.9% for IGFBP7. The intra-assay CVs were between 8.0 and 10.7% for TIMP-2 and 6.3 and 7.7% for IGFBP7.

The historical baseline serum creatinine concentration and glomerular filtration (eGFR) rate according to the Modification of Diet in Renal Disease (MDRD) equation were determined from available laboratory test results from the previous 12 months. Otherwise, a back-estimation of the serum creatinine concentration (sCr) was obtained with the MDRD equation, imputing an eGFR of 75 ml/min per 1.73 m^2^ [22, 23]. Urine output was collected via the bladder catheter, and baseline urine output was determined over the first 6 h of observation. The SCr was measured at least twice during the first 24 h and once daily thereafter. The kinetic eGFR was estimated from the change in SCr between inclusion and 24 h, with the creatinine clearance equation formulated by Chen et al. [19, 24]. The kinetic eGFR was not calculated for patients undergoing RRT at 24 h. Fluid overload was defined as a cumulative fluid balance exceeding 10% of body weight at admission [25].

AKI was defined according to the Kidney Disease Improving Global Outcome (KDIGO) classification as an increase in SCr of at least 26.5 μmol/l or to 1.5 times at baseline, or a urine volume < 0.5 ml/kg/h for 6 h. AKI severity was determined separately and was classified as mild (stage 1), moderate (stage 2), or severe (stage 3), based on the changes in creatinine concentration or urine output according to the KDIGO guidelines at inclusion, 24 h, 48 h, and 72 h. AKI was considered transient if kidney function was recovered within 3 days [26]. Recovery from AKI was defined as the disappearance of oliguria (in the absence of diuretic treatment) and/or a decrease in serum creatinine concentration of at least 50% and/or a return of serum creatinine concentration to the baseline value in the absence of RRT. For patients with both oliguria and SCr changes defining AKI, the correction of both plasma creatinine concentration and oliguria was for recovery to be considered to have occurred [9, 11]. Persistent AKI was defined as kidney dysfunction without recovery within 3 days or by the time of death, whichever occurred first.

### Data collection

Demographic data, such as age and sex, were recorded, together with body weight and any history of hypertension, diabetes mellitus, or chronic kidney disease. The origin of the sepsis was noted. During patient management, we noted whether patients had undergone imaging examinations with contrast agent injection (CT scan, coronary angiography), fluid therapy received within the first 48 h, vasopressor dose, the need for RRT, and the Simplified Acute Physiology Score (SAPS II) and the Sequential Organ Failure Assessment (SOFA) score assessed on admission to the ICU. The paraclinical data recorded included arterial lactate, blood urea nitrogen, and SCr levels. Mortality data were also recorded.

### Statistical analysis

Qualitative variables are expressed as the median [interquartile range (IQR)], and categorical variables are expressed as the frequency in absolute numbers (*n*) and as a percentage (%). Intergroup comparisons were performed with the Mann-Whitney *U* test, Fisher’s exact test, or the chi-squared test, as appropriate. Discriminative power was evaluated in a receiver operating characteristic (ROC) curve analysis. The area under the receiver-operating characteristic curve (AUROC) was recorded, together with its 95% confidence interval (CI). As recommended by Swets, an AUROC of 0.60–0.69 was defined as poor, 0.70–0.79 as fair, 0.80–0.89 as good, and 0.90–1.00 as excellent, in terms of predictive value [27, 28]. AUROCs were compared in DeLong’s test. We used the Youden index to determine the optimal cutoff for calculations of specificity, sensitivity, and positive and negative predictive performances. The Brier score was also calculated for each predictive parameter and model. Multivariable analysis was performed with a logistic regression model. We considered *p* < 0.05 to indicate statistical significance. All statistical analyses were performed with the MedCalc® software (version 17.9.7, MedCalc Software, Ostend, Belgium).

## Results

### Population characteristics

**Fig. 1:**
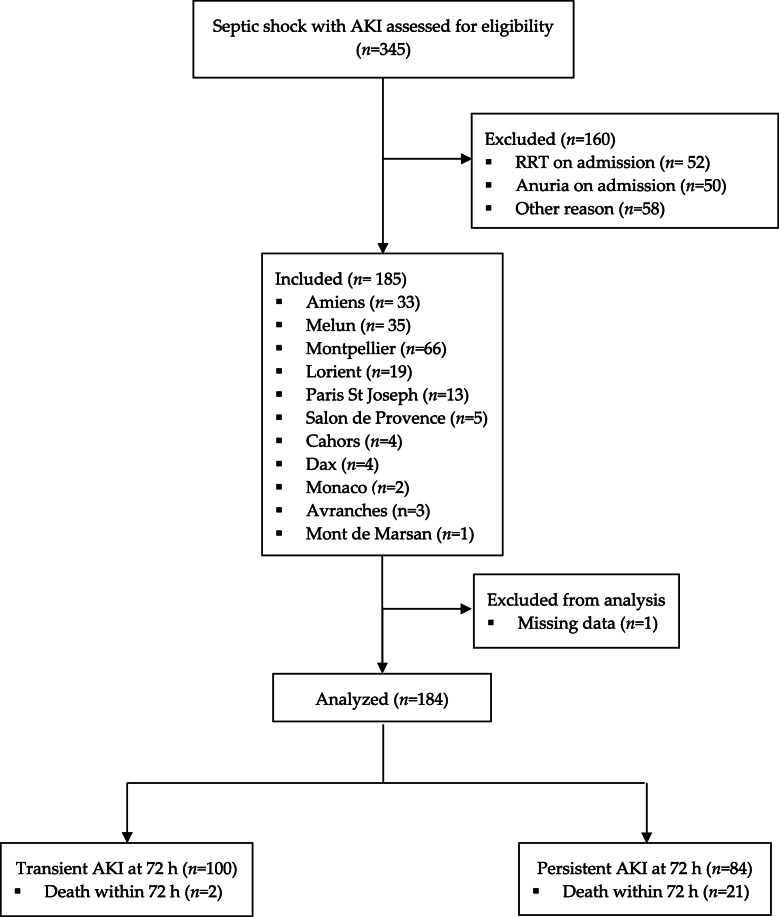
Study flow chart. RRT, renal replacement therapy; AKI, acute kidney injury

We screened 345 patients screened during the study period, 184 (166 (90%) at the five principal centers) of whom were included a median [IQR] of 1.0 [0.0–3.0] h after the initiation of norepinephrine treatment (Fig. 1). According to the definitions above, 84 (46%) patients had persistent AKI and 100 (54%) had transient AKI. The main characteristics of the study population are described in Table 1.

**Table 1:** Patient characteristics

	Total cohort (*n* = 184)	Transient AKI (*n* = 100)	Persistent AKI (*n* = 84)	*p* value
Demographic data				
Age (years)	69 [58–79]	67 [55–77]	71 [61–81]	0.1
Male	122 (66)	66 (66)	56 (67)	0.9
Hypertension	93 (49)	57 (57)	36 (43)	0.07
Diabetes mellitus	51 (28)	28 (28)	23 (27)	0.9
Congestive heart failure	15 (8)	6 (6)	9 (9)	0.2
Chronic kidney disease	23 (12)	10 (10)	13 (15)	0.3
Historical serum creatinine concentration (μmol/l)	80 [69–97]	77 [65–88]	87 [71–97]	0.03
Historical eGFR (ml/min per 1.73 m^2^)	77 [72–98]	79 [74–103]	76 [67–89]	0.03
Mode of admission				
Medical admission	168 (91)	92 (92)	76 (90)	0.1
Ward	48 (26)	22 (22)	26 (31)	0.2
Emergency room	82 (45)	48 (48)	34 (40)	0.3
Direct admission to ICU	30 (16)	20 (20)	10 (12)	0.1
Transferred from another center	24 (13)	10 (10)	14 (17)	0.2
Primary source of sepsis				
Pulmonary	105 (57)	58 (58)	47 (56)	0.8
Urinary	22 (12)	13 (13)	9 (11)	0.6
Intra-abdominal	37 (20)	17 (17)	20 (24)	0.2
Soft tissue	15 (8)	6 (6)	9 (11)	0.2
Germ identification	112 (61)	60 (60)	52 (62)	0.8
Gram-negative/Gram-positive	65/50	33/27	32/23	0.7
ICU management				
Time from vasopressor initiation to inclusion (h)	1.0 [0.0–3.0]	2.0 [0.0–3.5]	1.0 [0.0–3.0]	0.2
Perfusion fluid received before inclusion (ml/kg)	13.3 [5.8–21.0]	14.4 [5.1–19.8]	12.3 [5.9–23.2]	0.9
Contrast agent exposure before inclusion	36 (20)	20 (20)	16 (19)	0.9
Norepinephrine dose at inclusion (μg/kg/min)	0.36 [0.19–0.75]	0.32 [0.19–0.55]	0.46 [0.19–0.90]	0.05
SAPS II score	55 [42–69]	50 [39–60]	61 [50–75]	< 0.001
Baseline SOFA score	10 [8–12]	9 [8–11]	11 [9–13]	< 0.001
Baseline SOFA without renal component	8 [7–10]	8 [6–10]	9 [7–11]	0.01
Baseline lactate concentration (mmol/l)	2.0 [1.4–3.5]	1.8 [1.3–3.1]	2.6 [1.5–4.1]	0.007
Mechanical ventilation	101 (55)	50 (50)	51 (61)	0.1
Use of loop diuretics at 24 h	10 (5)	6 (6)	4 (5)	0.6
Perfusion fluid at 24 h (ml/kg)	33.3 [14.3–57.2]	31.5 [14.3–57.8]	33.8 [14.8–57.2]	0.7
Fluid balance at 24 h (ml/kg)	9.7 [− 12.7–33.1]	0.7 [− 18.8–21.7]	23.1 [− 1.5–49.3]	< 0.001
Fluid overload (> 10%) at 24 h	92 (50)	36 (36)	56 (67)	< 0.001
Norepinephrine dose at 24 h (μg/kg/min)	0.18 [0.04–0.72]	0.13 [0.02–0.36]	0.45 [0.09–1.15]	< 0.001
Fluid balance at 48 h (ml/kg)	0.8 [− 30.7–33.1]	− 17.6 [− 42.8–14.5]	22.1 [− 8.3–55.3]	< 0.001
Fluid overload (> 10%) at 48 h	78 (42)	29 (29)	49 (58)	< 0.001
Kidney function				
Baseline urine output (ml/kg/h)	0.59 [0.27–1.10]	0.83 [0.52–1.65]	0.31 [0.13–0.62]	< 0.001
Baseline serum creatinine concentration (μmol/l)	150 [111–216]	133 [107–178]	183 [120–239]	0.002
Baseline serum urea concentration (mmol/l)	13.7 [9.2–19.2]	12.7 [9.0–18.1]	15.0 [9.9–21.2]	0.1
Urine output at 24 h (ml/kg/h)	0.72 [0.34–1.33]	1.08 [0.65–1.51]	0.43 [0.06–0.84]	< 0.001
KDIGO stage at inclusion				0.01
Mild AKI	94 (51)	61 (61)	33 (39)	
Moderate AKI	55 (30)	24 (24)	31 (37)	
Severe AKI	35 (19)	15 (15)	20 (24)	
Serum creatinine concentration at 24 h (μmol/l)*	121 [86–180]	95 [75–125]	181 [140–258]	< 0.001
Kinetic eGFR (ml/min per 1.73 m^2^)*	55 [35–89]	76 [54–95]	28 [15–49]	< 0.001
Renal replacement therapy within 72 h	30 (16)	2 (2)	28 (33)	< 0.001
Outcome				
In-ICU mortality	55 (30)	14 (14)	41 (49)	< 0.001
Mortality at D28	54 (29)	14 (14)	40 (48)	< 0.001

Median and interquartile range, *n* (%)

*ICU* intensive care unit, *SOFA* Sequential Organ Failure Assessment, *SAPS II* Simplified Acute Physiology Score 2, *eGFR* estimated glomerular filtration rate

*Excluding patients on renal replacement therapy at 24 h

Demographic data and the source of infection were similar between the groups. Patients with persistent AKI had higher illness severity scores, such as median [IQR] SOFA score at inclusion (11 [9–13] vs. 9 [8–11], *p* < 0.001) and median [IQR] SAPS II (61 [50–75] vs. 50 [39–60], *p* < 0.001). At inclusion, 94 (51%) patients presented mild AKI, 55 (30%) presented moderate AKI, and 35 (19%) presented severe AKI. Those with persistent AKI presented more severe acute kidney injury than those with transient AKI, as reflected in their higher median [IQR] serum creatinine level (183 [120–239] vs. 133 [107–178] μmol/l, *p* = 0.002) and lower median [IQR] urine output (0.31 [0.13–0.62] vs. 0.83 [0.52–1.65] ml/kg/h, *p* < 0.001) at inclusion. The dose of norepinephrine administered during the first 24 h was higher in the persistent AKI group than in the transient AKI group. The volume of fluid infused at 24 h was similar between the groups, but the fluid balance was higher in the persistent AKI group.

### Follow-up at 72 h

**Fig. 2:**
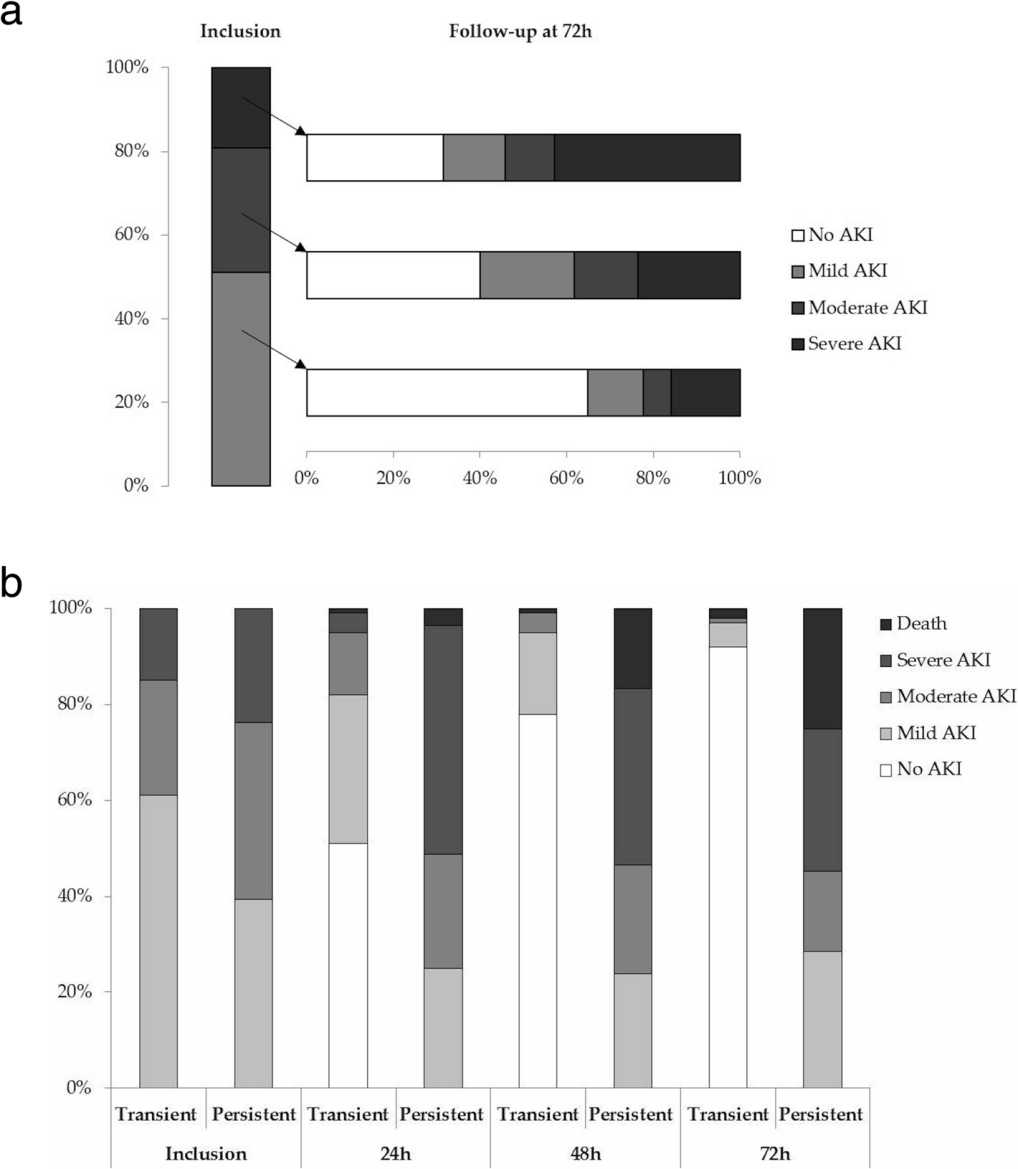
**a** Change in AKI severity over the first 72 h. The vertical bar indicates the AKI stage at inclusion according to the KDIGO classification. Horizontal bars indicate changes in the AKI stage over 72 h according to the AKI stage at inclusion. **b** Change in AKI severity and mortality in the transient and persistent AKI groups, over the first 72 h. The bars indicate the AKI stage and the proportion of patients dead at inclusion, 24 h, 48 h, and 72 h of follow-up

Patients presenting mild AKI at inclusion were more likely to display a complete recovery from AKI (65%) than patients with moderate (40%) or severe AKI (31%) (Fig. 2a). Twenty-eight (33%) patients in the persistent AKI group and only two patients in the transient AKI group received RRT (*p* < 0.001). Twenty-one (25%) patients in the persistent AKI group and only two (2%) in the transient AKI group died within the first 72 h (*p* < 0.001). Sixty-nine (55%) patients presented fluid overload within 72 h, including 32 with a urine [TIMP-2]*[IGFBP7] value H0 > 2.0. Twenty-six (81%) of these 32 patients presented persistent AKI, and 20 (62%) patients died within the first 72 h. The course of the disease over the first 72 h is presented for the transient and persistent AKI groups in Fig. 2b.

### Ability of [TIMP-2]*[IGFBP7] to predict persistent AKI

**Fig. 3:**
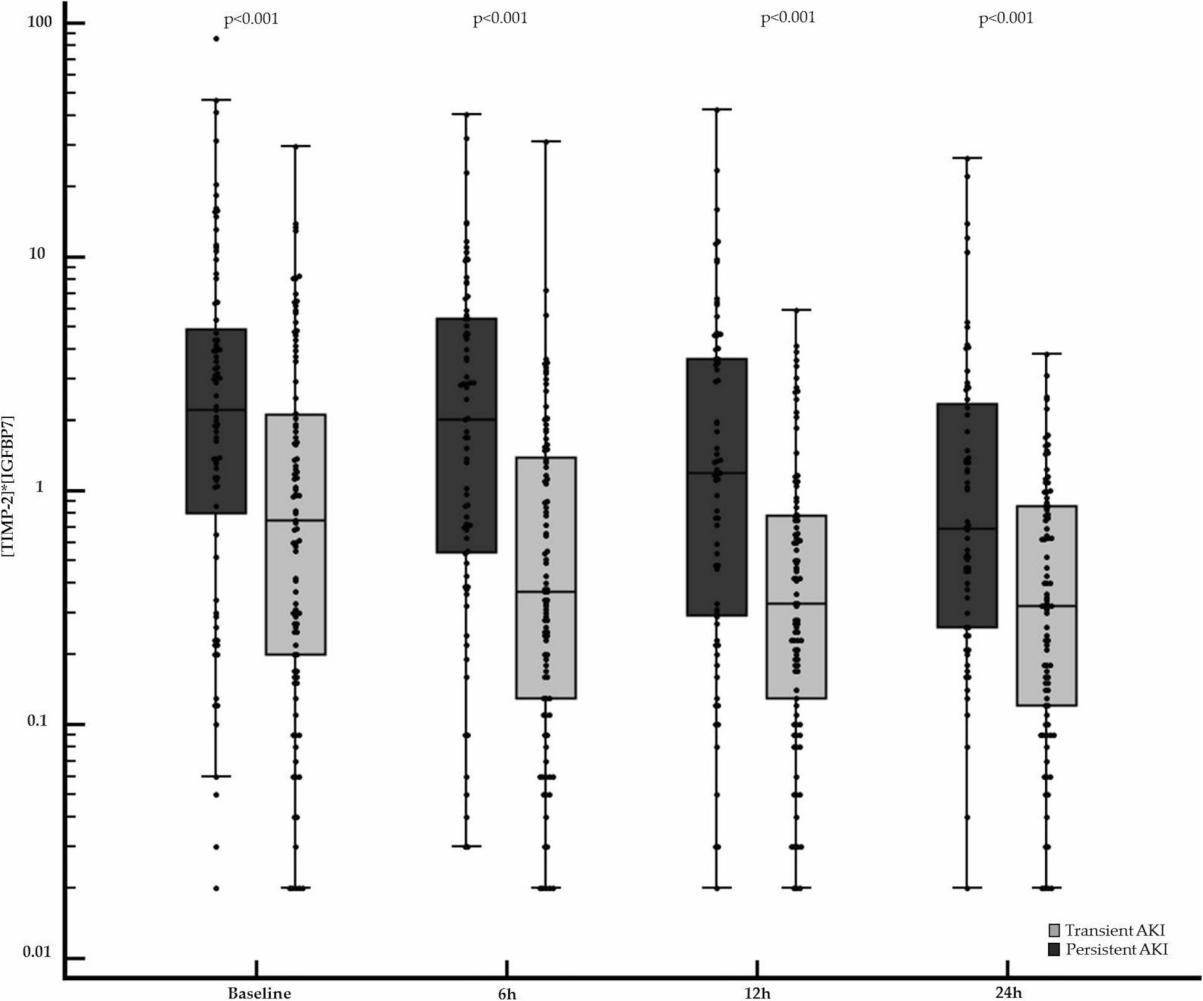
Box plots of the baseline, 6 h, 12 h, and 24 h urine [TIMP-2]*[IGFBP7] values. Log-transformed data area shown. The boxes and whiskers correspond to interquartile ranges and total ranges, respectively. Each dot represents an individual value. Urine [TIMP-2]*[IGFBP7] was significantly higher in patients with persistent AKI than in those with transient AKI. At baseline (*n* = 184), median [IQR] value of 2.21 [0.81–4.90] vs. 0.75 [0.20–2.12] (ng/ml)^2^/1000 (*p* < 0.001); at 6 h (*n* = 172), median [IQR] value of 2.02 [0.54–5.43] vs. 0.37 [0.13–1.38] (ng/ml)^2^/1000 (*p* < 0.001); at 12 h (*n* = 165), median [IQR] value of 1.19 [0.30–3.8] vs. 0.33 [0.13–0.78] (ng/ml)^2^/1000 (*p* < 0.001); and at 24 h (*n* = 156), median [IQR] value of 0.69 [0.26–2.36] vs. 0.32 [0.12–0.86] (ng/ml)^2^/1000 (< 0.001). TIMP-2, tissue inhibitor of metalloproteinases-2; IGFBP-7, insulin-like growth factor-binding protein 7; AKI, acute kidney injury; IQR, interquartile range

Urine [TIMP-2]*[IGFBP7] was significantly higher in the persistent AKI group than in the transient AKI group at H0, H6, H12, and H24 (Fig. 3). Urine [TIMP-2]*[IGFBP7] values tended to decrease within the first 12 h in both groups, but this trend was more marked during the first 6 h in the transient AKI group. At later time points (H12 to H24), TIMP-2*IGFBP7 tended to remain stable in both groups (Table 2).

**Table 2:** Variation of [TIMP-2]*[IGFBP7] at different time points

Parameters	Sample (*n*)	Total cohort	Transient AKI	Persistent AKI	*p* value
[TIMP-2]*[IGFBP7] at 0 h ([ng/ml]^2^/1000)	184	1.26 [0.26–4.00]	2.21 [0.81–4.90]	0.75 [0.20–2.12]	< 0.001
[TIMP-2]*[IGFBP7] at 6 h ([ng/ml]^2^/1000)	172	0.71 [0.22–2.86]	2.02 [0.54–5.43]	0.37 [0.13–1.38]	< 0.001
[TIMP-2]*[IGFBP7] at 12 h ([ng/ml]^2^/1000)	165	0.50 [0.19–1.59]	1.19 [0.30–3.8]	0.33 [0.13–0.78]	< 0.001
[TIMP-2]*[IGFBP7] at 24 h ([ng/ml]^2^/1000)	156	0.46 [0.16–1.18]	0.69 [0.26–2.36]	0.32 [0.12–0.86]	< 0.001
∆[TIMP-2]*[IGFBP7] 0 to 6 h (%)	172	− 20.0 [− 76.4–74.7]	− 36.5 [− 79.4–49.7]	11.3 [− 48.6–111.1]	0.04
∆[TIMP-2]*[IGFBP7] 0 to 12 h (%)	165	− 39.1 [− 77.2–56.4]	− 50.0 [− 84.0–50.0]	− 19.0 [− 70.6–100.4]	0.13
∆[TIMP-2]*[IGFBP7] 12 to 24 h (%)	156	0.0 [− 77.3–56.4]	0.0 [− 66.7–51.5]	3.7 [− 76.0–48.7]	0.99

Median and interquartile range

Baseline [TIMP-2]*[IGFBP7] was poorly discriminant, with an AUROC [95% CI] of 0.67 [0.59–0.73]. The highest Youden index was obtained for a baseline [TIMP-2]*[IGFBP7] level above 1.03 (ng/ml)^2^/1000, with a sensitivity of 74% [63–83] and a specificity of 59% [48–68]. The best discriminant performance of [TIMP-2]*[IGFBP7] was achieved at 6 h (172 samples available), with an AUROC [95% CI] of 0.73 [0.66–0.80]. A [TIMP-2]*[IGFBP7] level above 2.3 (ng/ml)^2^/1000 at 6 h predicted persistent AKI with a sensitivity of 48% [63–83] but a specificity of 88% [79–93] (Table 3). However, with an AUROC [95% CI] of 0.78 [0.71–0.83], baseline urine output performed as well as [TIMP-2]*[IGFBP7] at 6 h (*p* = 0.4) and better than baseline [TIMP-2]*[IGFBP7] (*p* = 0.02). A baseline urine output below 0.40 ml/kg/h was predictive of persistent AKI with a sensitivity of 62% [51–72] and a specificity of 88% [80–94]. The change in [TIMP-2]*[IGFBP7] between H0 and H6 (Δ0–6 h) or between H0 and H12 (Δ0–12 h) was not useful for predicting persistent AKI (Table 3).

**Table 3:** Performance of AKI markers for predicting persistent AKI

Parameters	Sample (*n*)	AUROC [95% CI]	Cutoff value	Younden index	Sensitivity [95% CI]	Specificity [95% CI]	PPV [95% CI]	NPV [95% CI]	Brier score^#^
[TIMP-2]*[IGFBP7] at 0 h ([ng/ml]^2^/1000)	184	0.67 [0.59–0.73]	> 1.03	0.33	74 [63–83]	59 [48–68]	59 [53–66]	73 [65–80]	0.23
[TIMP-2]*[IGFBP7] at 6 h ([ng/ml]^2^/1000)	172	0.73 [0.66–0.80]	> 2.3	0.36	48 [36–60]	88 [79–93]	75 [63–84]	68 [63–73]	0.21
[TIMP-2]*[IGFBP7] at 12 h ([ng/ml]^2^/1000)	165	0.70 [0.63–0.77]	> 1.1	0.39	56 [44–68]	83 [74–90]	71 [60–80]	72 [65–77]	0.21
[TIMP-2]*[IGFBP7] at 24 h ([ng/ml]^2^/1000)	156	0.68 [0.60–0.75]	> 0.43	0.27	67 [54–78]	60 [49–70]	54 [46–61]	72 [64–79]	0.21
∆[TIMP-2]*[IGFBP7] 0 to 6 h (%)	172	0.59 [0.51–0.67]	> − 55.6	0.22	78 [67–87]	44 [34–54]	51 [42–61]	72 [59–83]	0.24
∆[TIMP-2]*[IGFBP7] 0 to 12 h (%)	165	0.57 [0.49–0.65]	> − 67.1	0.16	74 [62–84]	42 [32–53]	49 [39–58]	68 [55–80]	0.24
Baseline urine output (ml/kg/h)	184	0.78 [0.71–0.83]	< 0.40	0.50	62 [51–72]	88 [80–94]	81 [71–88]	73 [67–78]	0.21
Urine output 6 to 12 h (ml/kg/h)	184	0.74 [0.67–0.80]	< 0.46	0.46	58 [47–69]	88 [80–93]	80 [69–87]	72 [66–77]	0.21
Urine output 12 to 24 h (ml/kg/h)	184	0.75 [0.68–.081]	< 0.47	0.42	54 [43–65]	88 [80–94]	79 [66–70]	61 [61–78]	0.22
SCr concentration at baseline (μmol/l)	184	0.63 [0.56–0.70]	> 179	0.29	52 [41–63]	77 [67–85]	66 [53–77]	65 [56–74]	0.24
SCr concentration at 24 h* (μmol/l)	160	0.84 [0.77–0.89]	> 139	0.60	79 [67–88]	81 [71–88]	75 [63–84]	84 [75–91]	0.16
Kinetic eGFR* (ml/min per 1.73 m^2^)	160	0.86 [0.80–0.91]	< 51	0.59	79 [67–88]	80 [71–88]	74 [63–84]	84 [75–91]	0.15

*AUROC* area under the receiver-operating characteristic curve, *TIMP-2* tissue inhibitor of metalloproteinases-2, *IGFBP-7* insulin-like growth factor-binding protein 7, *SCr* serum creatinine, *eGFR* estimated glomerular filtration rate, *PPV* positive predictive value, *NPV* negative predictive value

*Excluding patients undergoing renal replacement therapy at 24 h

^**#**^The maximum value expected is 0.25 given the prevalence of persistent AKI in the cohort (46%)

The discriminative performance of [TIMP-2]*[IGFBP7] was also assessed in the subgroup of 90 patients presenting moderate to severe AKI at inclusion, considering persistent AKI as a KDIGO stage ≥ 2 at 72 h. The performance of [TIMP-2]*[IGFBP7] remained similarly limited in this subgroup of patient (Supplemental Table 1).

### Combination of [TIMP-2]*[IGFBP7] with clinical data for the early prediction of persistent AKI

Clinical variables routinely available at a very early stage of care (in the first 6 h) for which significant differences were detected in the univariate analysis (baseline urine output, SOFA without a renal component, serum creatinine concentration, and dose of norepinephrine at inclusion) were combined in a multivariable logistic regression analysis, for the prediction of persistent AKI. The clinical prediction model had a good performance for predicting persistent AKI, with an AUROC [95% CI] of 0.81 [0.74–0.86] increasing to 0.86 [0.77–0.92] in the subgroup of patients presenting moderate to severe AKI at inclusion (Table 4). When combined with these parameters, baseline [TIMP-2]*[IGFBP7] was not independently associated with persistent AKI at 72 h and did not improve the performance of the clinical model for predicting persistent AKI.

**Table 4:** Logistic regression models for the early prediction of persistent AKI

Variable included	Clinical model		Model 2	
	Odds ratio [95% CI]	*p* value	Odds ratio [95% CI]	*p* value
Total cohort (*n* = 184)				
Baseline SOFA without renal component	1.16 [1.01–1.33]	0.02	1.16 [1.01–1.32]	0.03
Baseline urine output (ml/kg/h)	0.28 [0.16–0.49]	< 0.001	0.32 [0.19–0.56]	< 0.001
Baseline SCr concentration* (μmol/l)	1.30 [1.08–1.56]	0.005	1.30 [1.08–1.56]	0.006
Baseline [TIMP-2]*[IGFBP7]			1.04 [0.98–1.11]	0.2
Norepinephrine dose at inclusion (μg/kg/min)	2.75 [1.22–6.20]	0.01	2.67 [1.19–6.03]	0.02
AUROC	**0.81 [0.74–0.86]**		**0.81 [0.74–0.86]**	
Brier score^#^	**0.18**		**0.17**	
Moderate to severe AKI (*n* = 90)				
Baseline SOFA without renal component	1.37 [1.09–1.73]	0.008	1.39 [1.09–1.76]	0.007
Baseline urine output (ml/kg/h)	0.18 [0.07–0.46]	< 0.001	0.19 [0.07–0.49]	< 0.001
Baseline SCr concentration* (μmol/l)	1.22 [0.95–1.58]	0.1	1.23 [0.95–1.59]	0.1
Baseline [TIMP-2]*[IGFBP7]			1.01 [0.95–1.08]	0.7
Norepinephrine dose at inclusion (μg/kg/min)	4.55 [1.12–18.45]	0.03	4.32 [1.07–17.46]	0.04
AUROC	**0.86 [0.77–0.92]**		**0.86 [0.77–0.92]**	
Brier score^#^	**0.19**		**0.19**	

Data are presented as odds ratios [95% confidence intervals]. The population is divided into two groups according to AKI severity at inclusion

*TIMP-2* tissue inhibitor of metalloproteinases-2, *IGFBP-7* insulin-like growth factor-binding protein 7, *SOFA* Sequential Organ Failure Assessment, *SCr* serum creatinine

*For each 50 μmol/l increase

^**#**^The maximum value expected is 0.25, given the prevalence of persistent AKI in the cohort (46%)

## Discussion

The urine [TIMP-2]*[IGFBP7] values recorded in the early phase of septic shock were significantly higher in patients presenting persistent AKI than in those with transient AKI. However, given the wide overlap between the groups, these biomarkers discriminated poorly between the groups based on recovery from AKI and did not improve the prediction provided by the usual clinical variables. These results do not support the use of this marker to differentiate between transient and persistent AKI in the early phase of septic shock.

Both TIMP-2 and IGFBP7 are expressed in tubular cells exposed to cellular stress or injury, resulting in G1 cell cycle arrest, presumably to prevent potentially damaged cells from dividing [29]. Previous studies reported that urine [TIMP-2]*[IGFBP7] could be used for early diagnosis and risk prediction in sepsis-associated AKI. In a subgroup analysis of the Sapphire and Topaz studies [29, 30], urine [TIMP-2]*[IGFBP7] had an AUROC of 0.84 [0.73–0.92] for predicting moderate-to-severe AKI in a population of 232 septic patients [16]. More recently, in a population of 112 patients presenting with septic shock and mild-to-moderate AKI, we showed that, with an AUROC of 0.83 [0.75–0.90], urine [TIMP-2]*[IGFBP7] could be used to identify the subgroup of patients likely to display progression to severe AKI over the next 24 h [18].

Transient and persistent sepsis-associated AKI may have pathophysiological mechanisms in common [31], and it has been suggested that the reversibility of AKI is more related to the severity of kidney damage than to its mechanisms [32]. Urinary TIMP-2 and IGFBP7 concentrations are considered to be markers of kidney damage. We therefore hypothesized that urine [TIMP-2]*[IGFBP7] might be correlated with the duration of AKI. However, consistent with the findings of previous studies, our results do not support this hypothesis. Dewitte et al. assessed the performance of the urine [TIMP-2]*[IGFBP7] value obtained at ICU admission for predicting short-term recovery from AKI in a cohort of 56 unselected critically patients. Urine [TIMP-2]*[IGFBP7] had a fair performance, with an AUROC of 0.71 [0.57 to 0.82] [19]. In another single-center cohort of 41 patients, Daubin et al. showed that neither absolute values nor changes in [TIMP-2]*[IGFBP7] distinguished efficiently between transient and persistent AKI [20]. Contrary to our results, Daubin et al. found that median urine [TIMP-2]*[IGFBP7] was higher in the transient AKI group than in the persistent AKI group (0.87 vs. 0.13 (ng/ml)^2^/1000 *p* = 0.035), and their global absolute values were much lower than those observed here. Patients presenting with severe AKI or exposed to nephrotoxic agents before admission were not included in Daubin’s study, and 46% of the patients were not on vasopressive drugs at inclusion. We focused on septic shock patients, who are considered to be exposed to multiple kidney aggressions, potentially accounting for at least some of the differences between these studies. In a recent landmark study on the prediction of persistent AKI in critically ill patients (including 20% with sepsis), Hoste et al. found similar results to ours, with an AUC of 0.68 for TIMP-2*IGFBP7 for predicting persistent AKI [33]. The authors identified CCL14 as a new urine biomarker that seemed to reflect the extent of tissue damage rather than a specific mechanism of kidney lesions, as the expression of CCL14 was not influenced by the type of kidney damage (e.g., septic, nephrotoxic, inflammatory).

As previously reported, urine [TIMP-2]*[IGFBP7] tended to decrease in both groups over the first few hours. It is difficult to establish a link between the kinetics of these biomarkers and short-term changes in kidney function, because this value was not available for more severely affected patients who developed anuria or died soon after inclusion. The initial high [TIMP-2]*[IGFBP7] values in the persistent AKI group may reflect a stronger initial period of injury that did not persist for very long, potentially accounting for the progressive decrease in [TIMP-2]*[IGFBP7] in this group. Given the kinetics of these biomarkers, the wide range of values obtained may reflect the broad time window from the onset of kidney injury to the first urine sample, which is impossible to define in septic shock, contrary to other settings, such as cardiac surgery or cardiac arrest.

Patients who died within the first 72 h were considered to have transient or persistent AKI in accordance with the predetermined criteria. As expected, most of the early deaths occurred in the persistent AKI group. We cannot exclude the possibility that some of these patients might have recovered at later time points had they not died. However, a large database analysis established that recovery from AKI generally occurs within the first 3 days [26]. Moreover, the relationship between AKI duration and mortality is well established [34–36]*.* In a large database analysis, AKI recovery was found to be associated with a lower 28-day ICU mortality rate in patients with septic shock, with a cause-specific hazard ratio [95% CI] of 0.51 [0.41–0.64 [36].

The clinical model combining early illness and AKI severity markers was more effective for predicting short-term changes in kidney function than any parameter considered alone, particularly after the exclusion of patients presenting mild AKI, who were more likely to recover [3, 36]. Sepsis-associated AKI is a complex condition, and it is unlikely that any single marker of kidney injury will be able to predict fully the changes in kidney function. The integration and monitoring of usual parameters remains the most effective tool for guiding treatment decisions. For instance, the intensive monitoring of urine output is associated with better fluid management and a lower incidence of fluid overload in patients presenting AKI [37]. In our population, the larger proportion of patients presenting fluid overload in the persistent AKI group may also reflect the potentially deleterious effect of fluid accumulation on kidney function [5].

SCr was less discriminative than urine output. However, the distribution volume of serum creatinine is strongly influenced by capillary leakage and fluid accumulation, delaying the increase in SCr. In a recent study, de Jong et al. showed that combining SCr determination with multifrequency bioelectrical impedance analysis increased the predictive value of creatinine/urea clearance in critically ill patients, potentially improving the estimation of short-term kidney function changes in sepsis-associated AKI [38]. The combination of MBIA and cell cycle arrest may be synergistic, and this requires further investigations.

Patients without anuria, but requiring emergency RRT on admission, were not included in the study. We chose to focus on patients for whom the question of initiating RRT might be posed during ICU management. For the same reason, patients requiring emergency RRT were also not included in the recently published RUBY study either [33]. However, 35 of the patients studied here were placed on RRT within the first 72 h, including 19 within the first 24 h. [TIMP-2]*[IGFBP7] is correlated with the severity of kidney aggression. It therefore seemed likely that it would not differ significantly between patients presenting with severe AKI with and without indications for emergency RRT on admission.

No significant difference in comorbid conditions, such as chronic hypertension or diabetes, was observed between the two groups. This may seem surprising, but it may be due, at least in part, to the exclusion of patients presenting severe CKD. However, no major significant difference in comorbid conditions was reported in other larger similar cohorts [33, 36].

Our study was subject to several limitations. Variability in the timing of urine sampling may have affected urine [TIMP-2]*[IGFBP7] values, particularly as the time from kidney injury cannot be clearly determined in patients presenting with sepsis-associated AKI. The study was performed in 11 ICUs, but 90% of the patients were enrolled at five ICUs, with the other six ICUs enrolling no more than five patients each, as indicated on the flow chart for the study. This at least partly explains the limited number of patients (345 patients) screened during the study period, lower than would be expected for 11 recruiting centers, given the epidemiology of sepsis-associated AKI. Most of the patients included in this study (91%) were admitted for medical indications, and this may also limit the generalization of these results. Historical SCr levels were back-estimated for 61 patients, and this method may have led to the overdiagnosis or overstaging of AKI in some cases [39, 40]. However, excluding patients without prior SCr data would probably introduce a selection bias.

## Conclusion

In summary, we found that urine [TIMP-2]*[IGFBP7] measurements in the early phase of septic shock discriminated poorly between transient and persistent AKI and did not improve clinical prediction from the usual variables. This biomarker does not appear to be clinically useful for distinguishing between transient and persistent sepsis-associated AKI.

## Supplementary information

**Additional file 1: Supplemental Table 1.** Performance of AKI markers for predicting persistent AKI in patient presenting moderate to severe AKI at baseline.

## Data Availability

The datasets used and/or analyzed in this study are available from the corresponding author on request**.**

## Abbreviations

AKI: Acute kidney injury
AUROC: Area under the receiver-operating characteristic curve
CI: Confidence interval
CCL14: C-C motif chemokine ligand 14
eGFR: Estimated glomerular filtration rate
ICU: Intensive care unit
IGFBP7: Insulin-like growth factor-binding protein 7
IQR: Interquartile range
KDIGO: Kidney Disease Improving Global Outcomes
MBIA: Multifrequency bioelectrical impedance analysis
MDRD: Modification of Diet in Renal Disease
SAPS II: Simplified Acute Physiology Score
SCr: Serum creatinine
SOFA: Sequential Organ Failure Assessment
ROC: Receiver operating characteristic
RRT: Renal replacement therapy
TIMP-2: Tissue inhibitor of metalloproteinase-2

## References

[1] Uchino S, Kellum JA, Bellomo R, Doig GS, Morimatsu H, Morgera S (2005). Acute renal failure in critically ill patients: a multinational, multicenter study. JAMA..

[2] Bagshaw SM, Uchino S, Bellomo R, Morimatsu H, Morgera S, Schetz M (2007). Septic acute kidney injury in critically ill patients: clinical characteristics and outcomes. Clin J Am Soc Nephrol.

[3] Peters E, Antonelli M, Wittebole X, Nanchal R, François B, Sakr Y (2018). A worldwide multicentre evaluation of the influence of deterioration or improvement of acute kidney injury on clinical outcome in critically ill patients with and without sepsis at ICU admission: results from the Intensive Care Over Nations audit. Crit Care.

[4] Bouchard J, Soroko SB, Chertow GM, Himmelfarb J, Ikizler TA, Paganini EP (2009). Fluid accumulation, survival and recovery of kidney function in critically ill patients with acute kidney injury. Kidney Int.

[5] Legrand M, Dupuis C, Simon C, Gayat E, Mateo J, Lukaszewicz A-C (2013). Association between systemic hemodynamics and septic acute kidney injury in critically ill patients: a retrospective observational study. Crit Care.

[6] Prowle J, Bagshaw SM, Bellomo R (2012). Renal blood flow, fractional excretion of sodium and acute kidney injury: time for a new paradigm?. Curr Opin Crit Care.

[7] Calzavacca P, Evans RG, Bailey M, Bellomo R, May CN (2015). Cortical and medullary tissue perfusion and oxygenation in experimental septic acute kidney injury. Crit Care Med.

[8] Lankadeva YR, Kosaka J, Evans RG, Bailey SR, Bellomo R, May CN (2016). Intrarenal and urinary oxygenation during norepinephrine resuscitation in ovine septic acute kidney injury. Kidney Int.

[9] Darmon M, Vincent F, Dellamonica J, Schortgen F, Gonzalez F, Das V (2011). Diagnostic performance of fractional excretion of urea in the evaluation of critically ill patients with acute kidney injury: a multicenter cohort study. Crit Care.

[10] Dewitte A, Biais M, Petit L, Cochard J-F, Hilbert G, Combe C (2012). Fractional excretion of urea as a diagnostic index in acute kidney injury in intensive care patients. J Crit Care.

[11] Pons B, Lautrette A, Oziel J, Dellamonica J, Vermesch R, Ezingeard E (2013). Diagnostic accuracy of early urinary index changes in differentiating transient from persistent acute kidney injury in critically ill patients: multicenter cohort study. Crit Care.

[12] Bagshaw SM, Bennett M, Devarajan P, Bellomo R (2013). Urine biochemistry in septic and non-septic acute kidney injury: a prospective observational study. J Crit Care.

[13] Vanmassenhove J, Glorieux G, Hoste E, Dhondt A, Vanholder R, Van Biesen W (2013). Urinary output and fractional excretion of sodium and urea as indicators of transient versus intrinsic acute kidney injury during early sepsis. Crit Care.

[14] Wlodzimirow KA, Abu-Hanna A, NM RAA, Spronk PE, Hofstra LS, Kuiper MA (2014). Transient versus persistent acute kidney injury and the diagnostic performance of fractional excretion of urea in critically ill patients. Nephron Clin Pract.

[15] Maciel AT, Vitorio D (2017). Urine biochemistry assessment in critically ill patients: controversies and future perspectives. J Clin Monit Comput.

[16] Honore PM, Nguyen HB, Gong M, Chawla LS, Bagshaw SM, Artigas A (2016). Urinary tissue inhibitor of metalloproteinase-2 and insulin-like growth factor-binding protein 7 for risk stratification of acute kidney injury in patients with sepsis. Crit Care Med.

[17] Cuartero M, Ballús J, Sabater J, Pérez X, Nin N, Ordonez-Llanos J (2017). Cell-cycle arrest biomarkers in urine to predict acute kidney injury in septic and non-septic critically ill patients. Ann Intensive Care.

[18] Maizel J, Daubin D, Vong LV, Titeca-Beauport D, Wetzstein M, Kontar L (2019). Urinary TIMP2 and IGFBP7 identifies high risk patients of short-term progression from mild and moderate to severe acute kidney injury during septic shock: a prospective cohort study. Dis Markers.

[19] Dewitte A, Joannès-Boyau O, Sidobre C, Fleureau C, Bats M-L, Derache P (2015). Kinetic eGFR and novel AKI biomarkers to predict renal recovery. Clin J Am Soc Nephrol.

[20] Daubin D, Cristol JP, Dupuy AM, Kuster N, Besnard N, Platon L (2017). Urinary biomarkers IGFBP7 and TIMP-2 for the diagnostic assessment of transient and persistent acute kidney injury in critically ill patients. PLoS One.

[21] Singer M, Deutschman CS, Seymour CW, Shankar-Hari M, Annane D, Bauer M (2016). The Third International Consensus Definitions for Sepsis and Septic Shock (Sepsis-3). JAMA..

[22] Bellomo R, Ronco C, Kellum JA, Mehta RL, Palevsky P, Acute Dialysis Quality Initiative workgroup (2004). Acute renal failure - definition, outcome measures, animal models, fluid therapy and information technology needs: the Second International Consensus Conference of the Acute Dialysis Quality Initiative (ADQI) Group. Crit Care.

[23] Závada J, Hoste E, Cartin-Ceba R, Calzavacca P, Gajic O, Clermont G (2010). A comparison of three methods to estimate baseline creatinine for RIFLE classification. Nephrol Dial Transplant.

[24] Chen S (2013). Retooling the creatinine clearance equation to estimate kinetic GFR when the plasma creatinine is changing acutely. J Am Soc Nephrol.

[25] Claure-Del Granado R, Mehta RL. Fluid overload in the ICU: evaluation and management. BMC Nephrol. 2016;17(1):109. 10.1186/s12882-016-0323-6 PMC4970195 27484681

[26] Uchino S, Bellomo R, Bagshaw SM, Goldsmith D (2010). Transient azotaemia is associated with a high risk of death in hospitalized patients. Nephrol Dial Transplant.

[27] Swets JA (1988). Measuring the accuracy of diagnostic systems. Science..

[28] Haase-Fielitz A, Bellomo R, Devarajan P, Story D, Matalanis G, Dragun D (2009). Novel and conventional serum biomarkers predicting acute kidney injury in adult cardiac surgery--a prospective cohort study. Crit Care Med.

[29] Kashani K, Al-Khafaji A, Ardiles T, Artigas A, Bagshaw SM, Bell M (2013). Discovery and validation of cell cycle arrest biomarkers in human acute kidney injury. Crit Care.

[30] Bihorac A, Chawla LS, Shaw AD, Al-Khafaji A, Davison DL, Demuth GE (2014). Validation of cell-cycle arrest biomarkers for acute kidney injury using clinical adjudication. Am J Respir Crit Care Med.

[31] Langenberg C, Gobe G, Hood S, May CN, Bellomo R (2014). Renal histopathology during experimental septic acute kidney injury and recovery. Crit Care Med.

[32] Schneider AG, Bellomo R (2013). Urinalysis and pre-renal acute kidney injury: time to move on. Crit Care.

[33] Hoste E, Bihorac A, Al-Khafaji A, Ortega LM, Ostermann M, Haase M, et al. Identification and validation of biomarkers of persistent acute kidney injury: the RUBY study. Intensive Care Med. 2020;46(5):943-53. 10.1007/s00134-019-05919-0 PMC7210248 32025755

[34] Perinel S, Vincent F, Lautrette A, Dellamonica J, Mariat C, Zeni F (2015). Transient and persistent acute kidney injury and the risk of hospital mortality in critically ill patients: results of a multicenter cohort study. Crit Care Med.

[35] Sood MM, Shafer LA, Ho J, Reslerova M, Martinka G, Keenan S (2014). Early reversible acute kidney injury is associated with improved survival in septic shock. J Crit Care.

[36] Truche AS, Ragey SP, Souweine B, Bailly S, Zafrani L, Bouadma L (2018). ICU survival and need of renal replacement therapy with respect to AKI duration in critically ill patients. Ann Intensive Care.

[37] Jin K, Murugan R, Sileanu FE, Foldes E, Priyanka P, Clermont G (2017). Intensive monitoring of urine output is associated with increased detection of acute kidney injury and improved outcomes. Chest..

[38] de Jong LAA, Otten-Helmers AG, Spronk PE, van Kan HJM (2019). Bioelectrical impedance measurements for assessment of kidney function in critically ill patients. Crit Care Med.

[39] Siew ED, Matheny ME, Ikizler TA, Lewis JB, Miller RA, Waitman LR (2010). Commonly used surrogates for baseline renal function affect the classification and prognosis of acute kidney injury. Kidney Int.

[40] Siew ED, Matheny ME (2015). Choice of reference serum creatinine in defining acute kidney injury. Nephron..

