# Correction to: Urine cell cycle arrest biomarkers distinguish poorly between transient and persistent AKI in early septic shock: a prospective, multicenter study

**DOI:** 10.1186/s13054-020-03205-w

**Published:** 2020-08-04

**Authors:** Dimitri Titeca-Beauport, Delphine Daubin, Ly Van Vong, Guillaume Belliard, Cédric Bruel, Sami Alaya, Karim Chaoui, Maud Andrieu, Isabelle Rouquette-Vincenti, Frederic Godde, Michel Pascal, Momar Diouf, Christophe Vinsonneau, Kada Klouche, Julien Maizel

**Affiliations:** 1 BoReal Study Group, Medical Intensive Care Unit and EA7517, Amiens University Hospital, F-80054 Amiens, France; 2 Department of Intensive Care Medicine, Lapeyronie University Hospital, Montpellier, France; 3 Intensive Care Unit, Groupe Hospitalier Sud Ile de France, 270 avenue Marc Jacquet, 77000 Melun, France; 4 Medical-Surgical Intensive Care Unit, Centre Hospitalier de Bretagne Sud, Lorient, France; 5 Medical and Surgical Intensive Care Unit, Groupe Hospitalier Paris Saint Joseph, Paris, France; 6 Intensive Care Unit, Centre Hospitalier Général, 13300 Salon-de-Provence, France; 7 Intensive Care Unit, Jean Rougier Hospital, 335, rue du Président Wilson, 46000 Cahors, France; 8 Medical and Surgical Intensive Care Unit, Centre Hospitalier de Dax-Côte d’Argent, Dax, France; 9 Department of Anesthesia and Intensive Care, Princess Grace Hospital, Avenue Pasteur, Monaco (Principality), Monaco; 10 Département de Réanimation Polyvalente, Centre Hospitalier Avranches-Granville, Granville, France; 11 Intensive Care Unit, Centre Hospitalier de Mont De Marsan, 40000 Mont-de-Marsan, France; 12 Clinical Research and Innovation Directorate, Amiens University Hospital, Amiens, France; 13 BoReal Study Group, Intensive Care Unit, Hôpital de Bethune, 62408 Bethune, France

**Correction to: Crit Care 24, 280 (2020)**

**https://doi.org/10.1186/s13054-020-02984-6**

**Correction**

Following publication of the original article [1], the authors reported that Table 2 contained an error. The correct table is given below.

**Table 2:** Variation of [TIMP-2]*[IGFBP7] at different time points

**Parameters**	**Sample (*n*)**	**Total cohort**	**Transient AKI**	**Persistent AKI**	***p*-value**
[TIMP-2]*[IGFBP7] at 0 h ([ng/ml]^2^/1000)	184	1.26 [0.26–4.00]	0.75 [0.20–2.12]	2.21 [0.81–4.90]	< 0.001
[TIMP-2]*[IGFBP7] at 6 h ([ng/ml]^2^/1000)	172	0.71 [0.22–2.86]	0.37 [0.13–1.38]	2.02 [0.54–5.43]	< 0.001
[TIMP-2]*[IGFBP7] at 12 h ([ng/ml]^2^/1000)	165	0.50 [0.19–1.59]	0.33 [0.13–0.78]	1.19 [0.30–3.8]	< 0.001
[TIMP-2]*[IGFBP7] at 24 h ([ng/ml]^2^/1000)	156	0.46 [0.16–1.18]	0.32 [0.12–0.86]	0.69 [0.26–2.36]	< 0.001
∆ [TIMP-2]*[IGFBP7] 0 h to 6 h (%)	172	−20.0 [− 76.4–74.7]	11.3 [− 48.6–111.1]	−36.5 [− 79.4–49.7]	0.04
∆ [TIMP-2]*[IGFBP7] 0 h to 12 h (%)	165	− 39.1 [− 77.2–56.4]	−19.0 [− 70.6–100.4]	−50.0 [− 84.0–50.0]	0.13
∆ [TIMP-2]*[IGFBP7] 12 h to 24 h (%)	156	0.0 [−77.3–56.4]	3.7 [− 76.0–48.7]	0.0 [− 66.7–51.5]	0.99

Median and interquartile range

The original article [1] has been updated.
