# Supplementary material for: Urine cell cycle arrest biomarkers distinguish poorly between transient and persistent AKI in early septic shock: a prospective, multicenter study
Source: Crit Care. 2020 Jun 1;24:280. doi: 10.1186/s13054-020-02984-6 (PMC7268340; doi:10.1186/s13054-020-02984-6)
Supplement: Supplementary file 1 — Additional file 1: Supplemental Table 1. Performance of AKI markers for predicting persistent AKI in patient presenting moderate to severe AKI at baseline. [file 13054_2020_2984_MOESM1_ESM.docx]

| **Supplemental Table 1. Performance of AKI markers for predicting persistent AKI in patient presenting moderate to severe AKI at baseline.** | | | | | | | | |
| --- | --- | --- | --- | --- | --- | --- | --- | --- |
| **Parameters** | **Sample**  **(*n*)** | **AUROC**  **[95% CI]** | **Cutoff**  **value** | **Younden**  **index** | **Sensitivity**  **[95% CI]** | **Specificity**  **[95% CI]** | **PPV**  **[95% CI]** | **NPV**  **[95% CI]** |
| [TIMP-2]*[IGFBP7] at 0 h ([ng/ml]^2^/1000) | 90 | 0.64 [0.53− 0.74] | >1.37 | 0.26 | 72 [55− 85] | 54 [39− 68] | 55 [40− 69] | 71 [54− 85] |
| [TIMP-2]*[IGFBP7] at 6 h ([ng/ml]^2^/1000) | 86 | 0.77 [0.67− 0.85] | >3.52 | 0.42 | 50 [33− 67] | 92 [81− 98] | 82 [60− 95] | 72 [59− 82] |
| [TIMP-2]*[IGFBP7] at 12 h ([ng/ml]^2^/1000) | 83 | 0.81 [0.71− 0.81] | >1.2 | 0.51 | 67 [48− 82] | 84 [71− 93] | 73 [54− 88] | 79 [66− 89] |
| [TIMP-2]*[IGFBP7] at 24 h ([ng/ml]^2^/1000) | 77 | 0.78 [0.67− 0.87] | >0.23 | 0.43 | 89 [71− 98] | 54 [39− 68] | 51 [36− 66] | 90 [73− 98] |
| Baseline urine output (ml/kg/h) | 90 | 0.80 [0.71− 0.81] | ≤0.38 | 0.59 | 74 [58− 87] | 84 [71− 93] | 78 [62− 90] | 81 [68− 91] |
| AUROC, area under the receiver-operating characteristic curve; TIMP-2: tissue inhibitor of metalloproteinases-2; IGFBP-7: insulin-like growth factor-binding protein 7; SCr: serum creatinine; eGFR: estimated glomerular filtration rate; PPV: positive predictive value; NPV: negative predictive value. | | | | | | | | |
